# Behavior of light elements in iron-silicate-water-sulfur system during early Earth’s evolution

**DOI:** 10.1038/s41598-021-91801-3

**Published:** 2021-06-24

**Authors:** Riko Iizuka-Oku, Hirotada Gotou, Chikara Shito, Ko Fukuyama, Yuichiro Mori, Takanori Hattori, Asami Sano-Furukawa, Ken-ichi Funakoshi, Hiroyuki Kagi

**Affiliations:** 1 Geochemical Research Center, Graduate School of Science, The University of Tokyo, Tokyo, 113-0033 Japan; 2 Institute for Solid State Physics, The University of Tokyo, Kashiwa, Chiba 277-8581 Japan; 3 Neutron Science Section, Materials and Life Science Division, J-PARC Center, Japan Atomic Energy Agency, Tokai, Naka, Ibaraki 319-1195 Japan; 4 Cross Tokai Research Center for Neutron Science and Technology, Tokai, Naka, Ibaraki 319-1106 Japan; 5 Present Address: Geodynamics Research Center, Ehime University, Matsuyama, Ehime 790-8577 Japan

**Keywords:** Core processes, Geochemistry, Geophysics, Mineralogy

## Abstract

Hydrogen (H) is considered to be one of the candidates for light elements in the Earth’s core, but the amount and timing of delivery have been unknown. We investigated the effects of sulfur (S), another candidate element in the core, on deuteration of iron (Fe) in iron–silicate–water system up to 6–12 GPa, ~ 1200 K using in situ neutron diffraction measurements. The sample initially contained saturated water (D_2_O) as Mg(OD)_2_ in the ideal composition (Fe–MgSiO_3_–D_2_O) of the primitive Earth. In the existence of water and sulfur, phase transitions of Fe, dehydration of Mg(OD)_2_, and formation of iron sulfide (FeS) and silicates occurred with increasing temperature. The deuterium (D) solubility (*x*) in iron deuterides (FeD_*x*_) increased with temperature and pressure, resulting in a maximum of *x* = 0.33(4) for the hydrous sample without S at 11.2 GPa and 1067 K. FeS was hardly deuterated until Fe deuteration had completed. The lower D concentrations in the S-containing system do not exceed the miscibility gap (*x* <  ~ 0.4). Both H and S can be incorporated into solid Fe and other light elements could have dissolved into molten iron hydride and/or FeS during the later process of Earth’s evolution.

## Introduction

**Figure 1:**
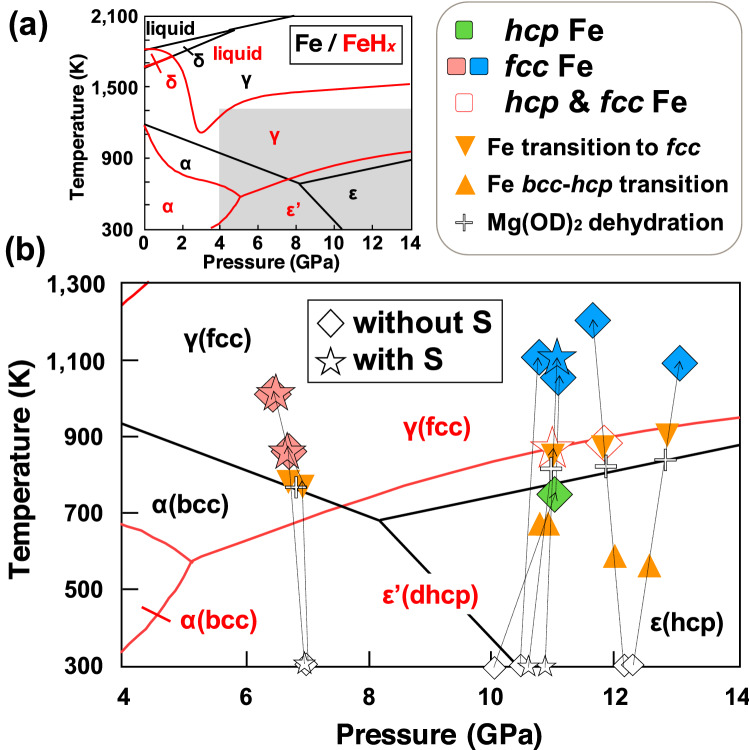
(**a**) Phase diagrams of iron and FeH_*x*_. Phase boundaries of FeH_*x*_ and Fe are from previous studies (Antonov et al.^30^; Fukai et al.^31^; Klotz et al.^32^). (**b**) An enlarged region of the gray area in (**a**) with PT paths of the neutron diffraction experiments. All data are hydrous samples with/without sulfur denoted as stars and diamonds, respectively. Larger symbols show the long-duration measurements. The occurrence of phase transitions of Fe is shown as triangles (filled triangle for transition to *fcc* from *bcc* or *hcp*; filled inverted triangle for *bcc*-*hcp* transition). The dehydration of Mg(OD)_2_ is denoted as “+” symbols.

Earth’s core currently consists of Fe with ~ 10% Ni and is considered to further contain several light elements, including H, C, O, Si, and S, in order to explain the density deficit compared with pure iron (10% for the outer liquid core, ~ 4% for the inner solid core; e.g.^1,2^). Specification of the light element(s) and verification of the evidence from seismological observations are long-standing research areas that have been addressed using high-pressure and high-temperature (high-PT) experiments or theoretical calculations. In particular, H, the lightest and most abundant element in the universe, is one of the most promising candidates of the light elements since it has been known that H solubility in Fe is significantly increased at high-P (e.g.^3^) and iron-water reaction was experimentally observed^4^. Only a small amount of H is required to strongly affect the density deficit and melting point depression of Fe. It is considered that the primitive atmosphere supplied a large amount of water vapor that was dissipated to the surface and transported into the Earth’s interior (e.g.^5^). A mixture of two components—i.e., ~ 90% of a low-melting-temperature component with a similar composition to carbonaceous chondrite (CI chondrite) and ~ 10% of a high-melting-temperature component, which is an aggregate of highly reduced, metal-rich devolatilized material similar in composition to enstatite chondrite—has been proposed as primordial material (e.g.^6^). Primordial materials should have resembled the constituents of the current bulk Earth in terms of elemental composition and isotope ratios. The building blocks that accreted to form the early Earth must have been highly reduced (e.g.^7^) and contained a substantial amount of water. CI chondrites are rich in water, up to 10 wt% (e.g.^8^), and thus 500–3000 ppm or more (1–10 wt%) water might have existed in the primordial materials during the accretion of Earth^5^. The depth of the magma ocean depends on the water content in the accreted planetesimals. As the amount of accreted water increased, the water content in the primitive atmosphere also increased owing to the strong thermal insulation involved in heating the surface, which caused the magma ocean to have a depth of ~ 1000 km (e.g.^9^). Core–mantle segregation probably proceeded in the presence of a large amount of water (or hydrogen). The solidus temperature of the core–mantle boundary would have decreased with the existence of H in the core^10^. However, the amount of H and the timing of its introduction remain unclear because of the experimental difficulties in observing the hydrogenation of Fe; iron hydrides are stable only under high-PT conditions and cannot be quenched at ambient pressure. H escapes easily from Fe by releasing pressure (e.g.^3^; see phase diagram in Fig. 1). In situ synchrotron X-ray observations have been conventionally performed to estimate the concentration of H (*x*) in iron hydrides (FeH_*x*_) from their volume increase by H dissolution^11–13^, although X-ray cannot directly detect atomic position and site occupancy of light elements (H) in the structure of such heavy metal hydrides. As the complimentary method of X-ray, in situ neutron diffraction observations have been recently applied to hydrogenation/deuteration of Fe under high-PT conditions^14–17^ using a combination of pulsed spallation neutrons and high-P apparatuses at the high-P beamline^18,19^ in J-PARC, Tokai, Japan. The volume increase per D (or H) atom ∆*V*(D/H) for iron deuterides/hydrides with face-centered cubic (*fcc*) structured and hexagonal close-packed (*hcp*) lattices have been also determined^14–16^.

Our previous study on the more complex Fe–hydrous silicate system clarified the amount of deuterium (D) in *fcc* Fe formed by the reaction with water (D_2_O) dehydrated from Mg(OD)_2_ as D source (Iizuka-Oku et al*.*^17^). In that study, we suggested that H dissolved preferentially into iron before other light elements had dissolved during the early stage of Earth’s evolution. In other work, Clesi et al*.*^20^ performed H partitioning experiments between Fe and unsaturated hydrous silicate melt, and argued that H is a lithophile element that can be incorporated readily into silicates. Those authors measured H concentrations in silicates and metals of quenched samples using elastic recoil detection analysis (ERDA), which showed that H in the metals was sufficiently low that the actual amount could not be determined after recovery. Their result is contrary to our previous results^17^ and to the results of quench experiments of rapidly decompressed samples obtained by Okuchi^21^, both of which indicated that H is a siderophile element and preferentially incorporated into Fe.

It is therefore important to further study the partitioning of light elements between solid iron hydride (not pure iron) and silicates through redox reactions. The present study focused on sulfur (S), a light element found in primitive meteorites and planetary cores. Among previous studies of the incorporation of H into the Fe–S system, dissolution of S into solid iron has been reported (e.g.^22,23^). Hydrogenation of FeS alloy starts at >  ~ 3 GPa and does not reach saturation (*x* = 0.2–0.4 for FeSH_*x*_^24,25^). This hydrogenation pressure is lower than that of FeSi alloy (> 10 GPa and *x* = 0.07–0.22 for FeSiH_*x*_^26^). The obtained H concentration in FeS is lower than that in pure iron but higher than that in FeSi. However, the values *x* were estimated from the volume increase of FeS high-PT phases (IV and V) using H-induced volume expansion ∆*V*(H) for the different materials. Several previous studies have conducted partitioning experiments on various systems, including Fe–O–Si, Fe–C–Si, Fe–C–S, and Fe–S–Si. For samples including H, the Fe–S–H^24,25^, Fe–Si–H^26^, Fe–H_2_O^27^, and Fe–C–H^28,29^ systems have been investigated. However, there have been no reports on the system including both silicates and H (i.e., the hydrous silicate system). Solid Fe can coexist with iron sulfide (FeS) at high-PT conditions, which lowers the melting temperature of iron. The eutectic temperature of the Fe–FeS system lies below the melting temperature of Fe, FeS, and iron hydrides (Shibazaki et al*.*^24^ and references therein). We therefore investigated the H concentration of iron in the Fe–hydrous silicate–S system using in situ neutron diffraction under high-PT conditions (6–12 GPa and up to ~ 1200 K) to clarify the effect of S on the hydrogenation of Fe and its implications for the evolution of the Earth. In all experiments of hydrous samples, a deuterated substitute Mg(OD)_2_ was used for water (D_2_O) source.

## Results

### Phase transitions of Fe and reactions at high PT

Table 1 summarizes initial compositions, products at high-PT, phases measured for a long duration at high-PT, and recovered phases. In the anhydrous system, which was examined for comparison of the water existence, the transition temperatures from body-centered cubic (*bcc*) to *hcp* and from *hcp* to *fcc* agreed with the phase diagram of pure iron^30–32^, and no deuteration (i.e., volume increase of Fe) occurred, as might have been expected; thus, the anhydrous samples are not discussed further here.

**Table 1:** List of experiments ordered by run number (Run#) and starting materials, involving the presence or absence of water and sulfur.

Initial sample	Run#	Sulfur content (wt%)	P (GPa)	T (K)	Observed Fe and FeS phases	Recovered species	
Fe, Mg(OD)_2_ + SiO_2_	A429	–	6.7	850	fcc	Fe, FeO	Fe-rich Ol, Fe-poor Px
			6.5	1000			
	A445		11	746	hcp		
			11.2	1067	fcc		
	A454		11.9	868	hcp, fcc		
			11.6	1177	fcc		
	A534		10.7	1116			
Fe + S, Mg(OD)_2_ + SiO_2_	A349	6	6.6	850	fcc, FeS-V	Fe, FeO, FeS-I	
			6.7	1000			
	A433	9	6.5	850			
			6.5	1000			
	A533	8	11.2	1116			
	A500*	9	11	~ 880	hcp, fcc, FeS-V		
Fe + S, MgO + SiO_2_	A430	7	6.6	850	fcc	Fe, FeO, FeS-I	En, SiO_2_
			6.4	1000			
Fe, MgO + SiO_2_	A453	–	11.8	700	hcp	Fe, FeO	
			11.4	1177	fcc		

The measurement of Fe and FeS phases was performed by neutron diffraction at high-PT conditions, and run products were identified from the recovered samples by SEM–EDS and XRD analyses. Symbols (star, diamond) for hydrous samples with/without S correspond to those presented in Figs. 1 and 3.

*Blowout occurred during the long-duration experiment in #A500. FeS–I (troilite) and FeS–V denote the ambient and Ni–As type of high-PT phases of iron sulfide, respectively. Observed silicates are olivine (Ol), pyroxene (Px), and/or enstatite (En). Uncertainties on pressures and temperatures during the long-duration experiments are ± 0.2 GPa and within 3 K, respectively (see main text).

In the hydrous system, dehydration of Mg(OD)_2_, which was loaded as an internal source of D through decomposition (i.e., Mg(OD)_2_ → MgO + D_2_O) accompanied with the redox reaction with Fe to form FeO, was observed as shown in the phase diagrams in Fig. 1. With increasing temperature at lower pressures (6–7 GPa), Fe transformed from *bcc* structure into *fcc* structure at ~ 800 K, where dehydration occurred almost simultaneously. At higher pressures (10–12 GPa), *bcc* Fe transformed into *hcp* at ~ 600 K and then into *fcc* at ~ 850 K, followed by dehydration of Mg(OD)_2_ at ~ 800 K. After the dehydration of Mg(OD)_2_, the unit cell volume of *fcc* or *hcp* Fe started to increase as a result of the deuteration. The phase boundaries determined for *hcp*–*fcc* Fe agreed well with those of previous studies (Fig. 1).

The temperature range where the single phase of *hcp* Fe was observed at around 10–12 GPa (Fig. 1) in the hydrous system was quite narrow because the *hcp* phase existed mostly together with the other Fe polymorphs (*bcc* or *fcc*). As the dehydration temperature of Mg(OD)_2_ increases with pressure and overlaps with the stability field of *fcc* Fe, deuteration of the single *hcp* Fe phase was not observed. Deuterated *hcp* and *fcc* Fe phases were observed in some experimental runs. After quenching to room temperature, *fcc* Fe partly back-transformed to *bcc* Fe at ~ 6 GPa, while *fcc* Fe remained together with *hcp* Fe at pressures of ~ 10 GPa. The single phase of *hcp* Fe could not be obtained during the cooling probably because of the slow kinetics of the phase transformation and the persistence of other phases as thermodynamically metastable states. No double-hexagonal close-packed (*dhcp*) phase (FeD_1.0_) was expectedly observed during heating or cooling paths, likely because the D pressures were not sufficiently high for Fe to achieve the saturated D concentration (as discussed below).

**Figure 2:**
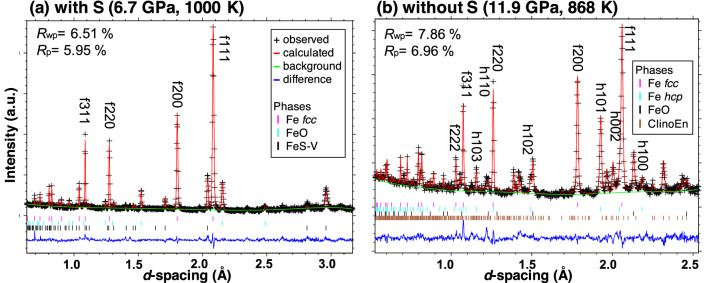
Representative neutron diffraction patterns analyzed using the Rietveld refinement method. (**a**) Hydrous sample with sulfur at 6.7 GPa and 1000 K (#A349) and (**b**) hydrous sample without sulfur at 11.6 GPa and 868 K (#A454). “+ ” denotes the observed data points; the solid line red denotes the calculated profile. The difference between the observed (black+) and the calculated (red) profiles (“diff”) is shown as blue lines at the bottom of each plot. Tick marks below the pattern show the positions of allowed diffraction peaks and *hkl* for the corresponding phases (f; Fe *fcc*, h; Fe *hcp*). “Clino En” represents the high-PT phase of enstatite.

For samples including S, an Ni–As type structured FeS (FeS–V phase; hereafter as FeS) formed in the high-PT region where *fcc* Fe was stable (stability field of FeS is not shown in Fig. 1; see diffraction patterns in Fig. 2a). The formation of the FeS phase was in good agreement with the phase diagram of FeS reported by Urakawa et al*.*^33^. Our preliminary quenching experiments and synchrotron X-ray observations revealed that Fe and FeS coexisted in the sample initially containing S. The eutectic temperature of the Fe–FeS binary system is below 1200 K, which is lower than the melting temperatures of pure Fe and hydrides. Neutron diffraction patterns and textures of the recovered samples indicated that the whole sample was solid during the long-duration measurements (as discussed below).

### Site occupancies and atomic volume of deuterium

The site occupancies of D in the crystal structure of FeD_*x*_ obtained using Rietveld analysis are listed in Supplementary Table S1. Figure 2 shows diffraction patterns of hydrous samples (a) initially including S and (b) without S, respectively, after the Rietveld refinement. Minor amounts of silicates (olivine and/or enstatite) surrounding the Fe (described below) were observed because they were hit by the tail of the incident beam profile. In the hydrous sample initially including S (#A349 in Fig. 2a) at 6.7 GPa and 1000 K, the dissolved D in *fcc* FeD_*x*_ was found in both octahedral and tetrahedral sites with occupancies of *g*(O) = 0.06(2) and *g*(T) = 0.03(1), respectively, resulting in a D concentration of FeD_*x*_ of *x* = *g*(O) + 2 × *g*(T) = 0.12(2). The coexisting FeS did not contain any noticeable amount of D, whose *x* values were negative or *x* = 0. At 12.0 GPa and ~ 900 K, both *fcc* and *hcp* FeD_*x*_ were observed in the sample without S (#A454 in Fig. 2b). The site occupancies of D in *fcc* FeD_*x*_ were *g*(O) = 0.19(1) and *g*(T) = 0.03(1), leading to *x* = 0.25(2). The *hcp* FeD_*x*_ contained less D (*x* = 0.10(2)) relative to *fcc* FeD_*x*_. Iron oxide (FeO) was observed in the hydrous sample, which formed via redox reaction between Fe and D_2_O supplied from Mg(OD)_2_ according to the following reaction^17^:

1 $${\text{2Fe }} + {\text{ D}}_{{2}} {\text{O}} \to {\text{FeO }} + {\text{ FeD}}_{x} + \, \left( {{1 } - x/{2}} \right){\text{ D}}_{{2}} .$$

FeO is expelled gradually from the above redox reaction by forming Fe-rich olivine. No diffraction peaks assignable to solid D_2_ were detected, suggesting that D_2_ produced from the reaction of Eq. (1) is fluid and was incorporated rapidly into Fe or might have escaped from the graphite inner capsule as a crystalline phase.

Machida et al*.*^14,15^ conducted high-PT experiments on the Fe–D system and determined the site occupancy of D, and ∆*V*(D) values for *fcc* and *hcp* FeD_*x*_ [2.21 ± 0.04 Å^3^ and 2.48 ± 0.05 Å^3^, respectively]. Ikuta et al*.*^16^ examined *fcc* FeH_*x*_ under similar experimental conditions to those of Machida et al*.*^14^ and reported a similar value of ∆*V*(H) = 2.22 ± 0.36 Å^3^ to that of *fcc* FeD_*x*_. The D concentrations and volume increase of *fcc* FeD_*x*_ calculated in the present study (∆*V*(D) = 2.29 ± 0.67 Å^3^) were close to the above values reported by previous neutron experiments^14,16^).

### Temperature dependence of deuterium concentration

**Figure 3:**
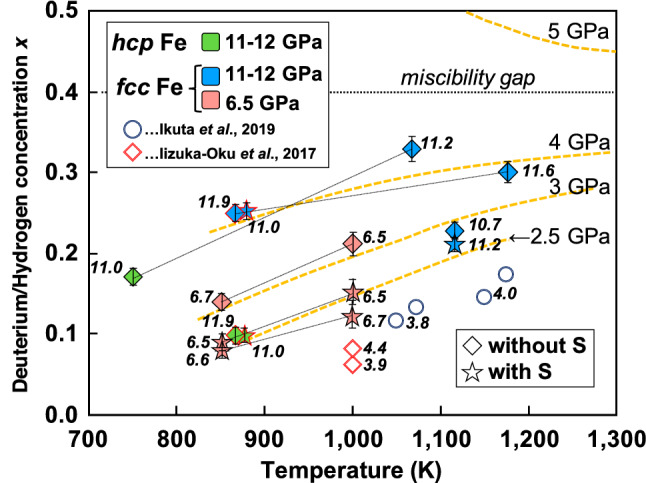
Plots of deuterium/hydrogen concentration (*x*) versus T for *hcp* and *fcc* Fe phases. Pressure values in GPa are shown on each plot. Symbols and colors are the same as those in Fig. 1. Data from previous studies (blue circles from Ikuta et al*.*^16^ and red diamonds from Iizuka-Oku et al*.*^17^) are also plotted. Dotted yellow lines show P–*x*–T relations in the Fe–H system of Hiroi et al*.*^34^. The miscibility gap they reported is shown at *x* ~ 0.4 as a dotted horizontal line.

Figure 3 shows a summary of the concentration of D dissolved into Fe and the PT relations obtained from all data. Some uncertainties in D concentration and site occupancies were as large as the last digit (Supplementary Table S1) in this study because the analyses were performed on multiple phases, including FeS, FeO, and silicates.

For iron deuterides synthesized in the present study, the obtained D concentrations shown in Fig. 3 were lower than those in previous studies of Fe–D or Fe–H systems^14–16^, regardless of the existence of S. This is because in our Fe–hydrous silicate system, the deuteration process through the redox reaction expressed by Eq. (1) is slow, and the D pressure was not high enough to supply D to Fe, even though the system initially contained saturated water (D_2_O). The solubility of D increased with increasing temperature for pressures of both 6–7 GPa and 10–12 GPa, suggesting that the dissolution of D into *fcc* Fe (i.e., the reaction expressed by Eq. (1)) is an endothermic process. The solubility of D was also positively correlated with pressure. This trend is in good agreement with the results for FeH_*x*_ with a lower H concentration in the Fe–H system reported by Hiroi et al*.*^34^, in which the dissolution of H into *fcc* Fe increases with temperature (i.e., the endothermic reaction of Fe + *x*/2 H_2_ → FeH_*x*_). According to the theoretical calculations of those authors, a miscibility gap was found at *x* =  ~ 0.4 between the regions of lower and higher H (D) concentrations. The concentrations of D of the present study were much lower than the values expected at similar PT conditions (around 0.6–0.7 at 6–7 GPa and 1000 K) for the Fe–H system^34^ and were located below their miscibility gap. The maximum solubility of D obtained in the present study was *x* = 0.33(4) for the hydrous sample without S (#A445) at 11.2 GPa and 1067 K. As the solubility of D unlikely exceeds *x* =  ~ 0.4, even when extrapolated to much higher-PT conditions, the highest concentration of H in the Fe–silicate–water system lies below this miscibility gap. In contrast, Shibazaki et al*.*^13^ showed that the concentration of H in *fcc* FeH_*x*_ was almost saturated (*x* =  ~ 1 or more) at 17–21 GPa and 1273 K. The rate of the hydrogenation was higher in that study (equilibrium was reached in 30 min) compared with our study. Those authors conducted in situ synchrotron X-ray experiments on almost the same hydrous system (Fe:Mg(OH)_2_:SiO_2_ = 2:1:1 in molar ratio, initially containing a water (H_2_O) content of ~ 8 wt%) as our samples without S. The difference is probably because of the higher-PT condition of their experiments, the difference in the initial sample volume (less than half of ours), sample loading conditions (fully mixed powder), and/or the different use of high-pressure devices and sample capsules (boron nitride).

### The effect of sulfur on deuteration

The concentration of D in Fe in the S-containing system (data shown as stars in Fig. 3) is slightly lower than that without S (data shown as diamonds) at any temperatures (850 K, 1000 K, and ~ 1100 K). This indicates that S in the system tends to suppress deuteration of Fe. In contrast, FeS did not incorporate D at all: a value of *x* = 0 or a negative value was obtained from the Rietveld analysis. This is consistent with the results that the unit cell volume of FeS was constant within errors regardless of temperature and time (for ~ 10 h; Supplementary Fig. S1), in agreement with those previously reported for the low-P phase (LPP) or high-P phase (HPP) of pure FeS at corresponding PT conditions^33^. Shibazaki et al*.*^24^ theoretically estimated that the most stable sites for H atoms in FeS with a structure similar to that of *dhcp* Fe are octahedral. Those authors reported that the maximum concentration of H in FeSH_*x*_ was *x* =  ~ 0.2 according to the volume increase from pure FeS. These differing results from those of the present study suggest that the hydrogenation (deuteration) of FeS was negligible in the Fe–hydrous silicate system, especially under the lower pressure of H (D). In the system without silicates that those authors investigated (the FeS–H system), hydrogenation of single-phase FeS would be possible^24^ but would be impossible in the present system in which Fe and FeS coexist and deuteration occurs via D_2_O. We also propose that in the range of lower D concentration considered in the present study, the deuteration of FeS must be negligible until the deuteration of Fe has been completed.

### Analyses of recovered samples

**Figure 4:**
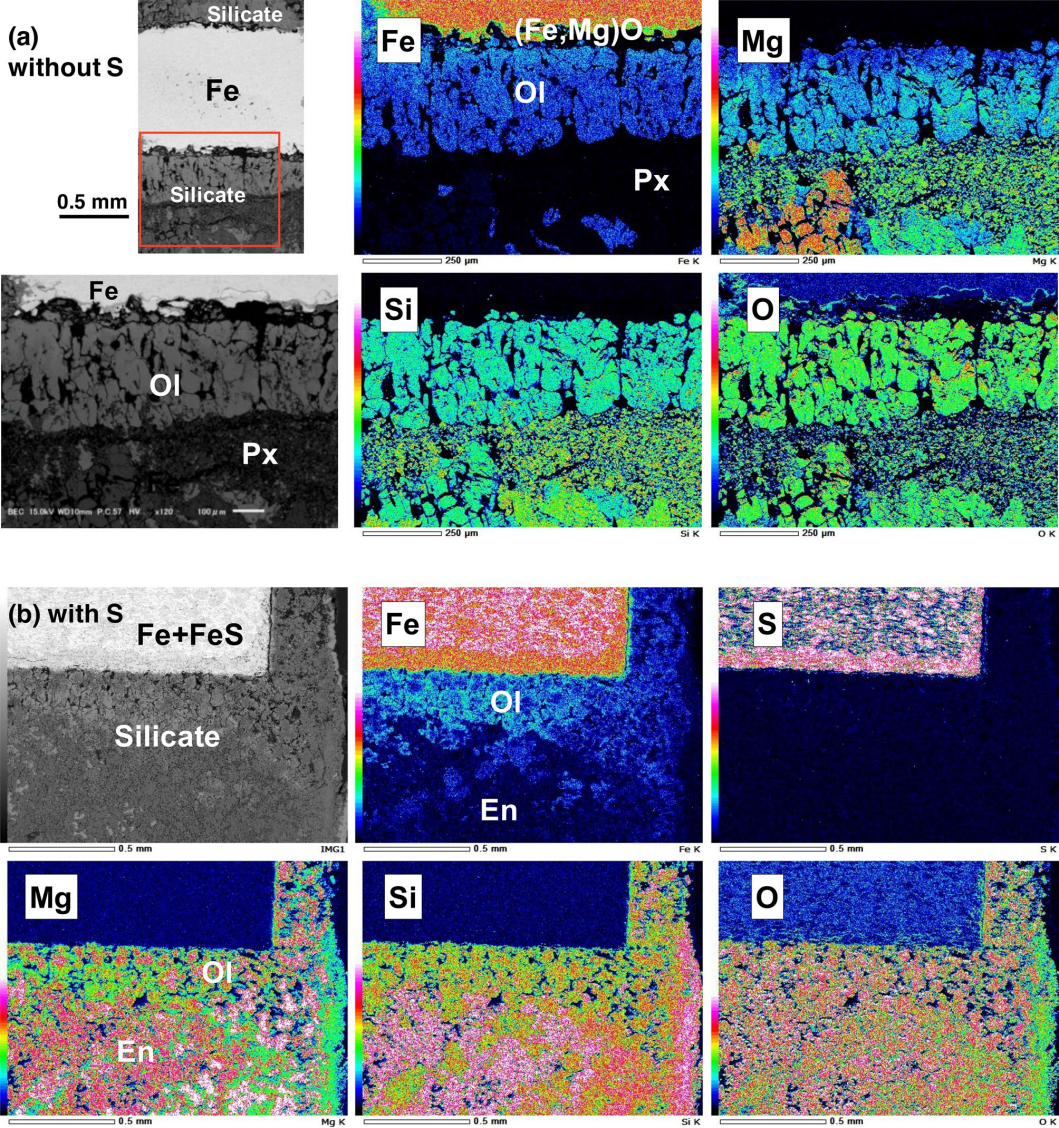
SEM elemental maps (Fe, Mg, Si, O and S) of hydrous samples, (**a**) without S recovered at 10.7 GPa and 1116 K (#A534) of the area surrounded by a red square in the upper left electron image and (**b**) with S recovered at 6.5 GPa and 1000 K (#A433). *Ol* olivine (Mg, Fe)_2_SiO_4_; *Px* pyroxene (Mg, Fe)SiO_3_; *En* enstatite MgSiO_3_. Olivine containing rich Fe located close to Fe whereas pyroxene was poor in Fe almost like enstatite and located far from the iron.

Figure 4 shows elemental mapping results for the recovered hydrous samples. In the S-free hydrous samples (Fig. 4a), Fe was surrounded by a layer of FeO [more precisely, (Fe_0.94–0.96_, Mg_0.06–0.04_)O], which was similar to that observed in our previous study^17^ and formed by the redox reaction (Eq. (1)) of Fe and water (D_2_O). We also observed Fe-poor pyroxene (Px) and Fe-rich olivine (Ol), which were likely formed by the following reactions (Eqs. (2), (3)^4^):

2 $${\text{MgO }} + {\text{ SiO}}_{{2}} \to {\text{MgSiO}}_{{3}} \left( {{\text{Fe-poor Px}}} \right),$$

3 $${\text{FeO }} + {\text{ MgSiO}}_{{3}} \to \left( {{\text{Fe}},{\text{ Mg}}} \right)_{{2}} {\text{SiO}}_{{4}} \left( {{\text{Fe-rich Ol}}} \right).$$

These reactions are consistent with the observation of the present study that enstatite formed at lower T and changed into olivine at higher T by reaction with FeO, which was yielded by the oxidation of Fe. The amount of Fe in olivine (Fe/(Fe + Mg) < 1.7) decreases with increasing distance from metallic Fe. A reaction gradient accompanied by the slow diffusion of Fe into olivine appeared to occur for the sample initially including D_2_O. The anhydrous sample included MgSiO_3_ enstatite and a small amount of SiO_2_ as residue, but no olivine, in line with the enhancement of transport of Fe into silicates by D_2_O. Neither Fe nor silicates were contaminated by carbon from the sample capsule at long as both phases remained solid.

The sample that initially included S and was recovered from 6.5 GPa and 1000 K (#A433) contained FeS–I (troilite), which was well mixed with Fe in the initial Fe + S pellet (Fig. 4b). No S was observed in silicates, indicating that S is a siderophile element and reacts only with Fe to form FeS. This suggests that both S and Fe can be mobile and that their reaction is promoted by water. Fe can also be incorporated into olivine only in the presence of water.

**Figure 5:**
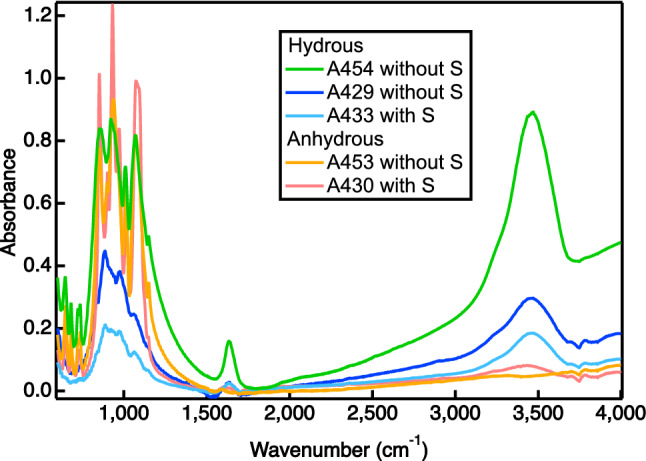
Infrared absorption spectra of recovered samples. The OH stretching bands at ~ 3450 cm^−1^ were observed only for the hydrous samples.

Figure 5 shows IR absorption spectra of all samples in the present study. A broad absorption band at ~ 3450 cm^−1^ assignable to the OH stretch mode of molecular H_2_O was detected only for hydrous samples. No peaks related to OD or D_2_O were observed. The complete substitution between H and D cannot easily takes place over time (several months to years) after recovery. The most likely source of H_2_O is derived from remaining absorbed water on the surface of the numerous cracks of silicates. That leads to a difficulty in achieving full quantification of water in silicates, especially for the hydrous samples, after a double-sided polish. No observation of D_2_O or OD in spectra indicates that the amount of D in silicates is below the detection limit and most D was partitioned into iron, not into silicates.

## Discussion: implications for the early Earth’s evolution

The present study investigated samples containing all possible light elements in the studied system, including H, O, Si, and S (C from the sample capsule is excluded and not considered hereafter), and revealed that both H and S are preferentially incorporated into solid Fe to be stabilized as FeH_*x*_ and FeS before melting. When Fe incorporates H to form solid solutions or hydrides, the hydrogenation of Fe is suppressed by S (FeS). There seemed to be no preference for the reaction of Fe with H or S. In addition, these two light elements substantially lower the melting temperature of the Fe(H_*x*_)–FeS(H_*x*_) system (Shibazaki et al.^24,25^), which promotes dissolution of the other light elements into molten iron. Therefore, the primordial Earth could have accommodated H and S into solid iron to be molten in the very early stages of Earth’s evolution during the accretion of planetesimals. The molten FeH_*x*_ and/or FeS might then have become separated from the bottom of the magma ocean and gradually sank to the core while incorporating the other light elements.

The primordial source of water and the timing of its delivery are still unknown (e.g.^35,36^), but it is likely that water and volatile elements were delivered during the early stage of Earth’s evolution (during the accumulation of planetesimals) rather than a much later stage of evolution. The depth of the magma ocean is thought to have been 700–1000 km (roughly 40–60 GPa) and its temperature less than 3000–3500 K when it contained abundant water^37^. The amount of water has yet to be determined, but it has been estimated to be over 700 ppm on the basis of several models. Clesi et al*.*^20^ experimentally reported that the amount of water in silicates was around 400–750 ppm. Oxygen fugacity (*f*O_2_) is also an important factor to constrain for primitive Earth conditions, but the details are not discussed here because Fe and silicates are solid, not melt in the present study. Ikuta et al*.*^16^ proposed that the concentration of H is 0.65 ± 0.25 wt% in Earth’s outer core and 0.12 ± 0.05 wt% in the inner core after correction of previous estimations by Narygina et al*.*^28^ and Thompson et al*.*^38^. Our results show that the upper limit of D that is dissolved into solid Fe is *x* =  ~ 0.4 for the studied Fe–hydrous silicate system, in which the pressure of H is substantially lower than that of previous studies. This leads to a value of ~ 0.35 wt% of H in iron hydrides if D is substituted completely by H, which is in agreement with the previous estimations described above.

Considering the effect of Ni, which constitutes 10 wt% of the current core, Shibazaki et al*.*^39^ reported hydrogenation of Fe–Ni alloy with an exothermic reaction at ~ 3 GPa, which is contrary to the endothermic reaction for Fe of the present study. Nickel is a 3d transition metal and utilizes H to decrease the enthalpy of solution, which shifts the miscibility gap toward a lower concentration of H^40^. Exothermic behavior in the *fcc* Ni–H system can be interpreted as the existence of a miscibility gap at around 1–2 GPa, and this pressure is lower than that at which the gap appears in pure Fe (4–5 GPa)^41^. Hydrogenation through the redox reaction did not exceed the miscibility gap of *x* =  ~ 0.4 in the present study. Therefore, the miscibility gap of Fe–Ni alloy in the hydrous system is expected to shift to much lower H concentrations. Ohta et al*.*^42^ measured the electrical resistivities of *fcc-*structured Fe and Fe–Ni hydrides and reported that they are fairly similar to each other because the effects of Ni and H are canceled out.

The existence of H would affect the concentration of other light elements in the core, as suggested by several studies of the various systems (e.g., the present study for Fe–silicate–H_2_O–S; Shibazaki et al*.*^24^ for FeS–H; Terasaki et al*.*^26^ for FeSi–H; and Hirose et al*.*^29^ for Fe–C–H). Further discussion of the current core, especially the outer core, will require studies of liquid metal at much higher PT conditions because the H concentration in liquid metal has been experimentally estimated based on properties of the solid phase. Although liquid Fe–H alloy could exist, as suggested by first-principle molecular dynamic calculations^43^, the absolute amount of H incorporated in liquid Fe is still unknown. In situ neutron diffraction of liquid Fe under high-PT conditions is one of the best solutions for determining its concentration of H, but is still a difficult approach mainly because of the following experimental challenges: (1) there is the problem of maintaining liquid at high temperatures (above 1500 K) for a long time to obtain diffraction patterns; (2) analyzing the diffraction patterns of the liquid phase at much higher-PT are more difficult (but challenging) than those of the solid phases; and (3) it would be preferable and easier to determine the concentration of H in quenched melt samples, but the effect of grain growth on diffraction intensity should be carefully considered and most of H might be escaped after quench. Therefore, there is a need to improve the technique of rapid quenching into liquid nitrogen as applied previously by Antonov et al.^44^. More than 95% of water reacts with Fe at 7.5 GPa, and molten iron can incorporate a larger amount of H than can solid iron^21^, but the amount of H in iron will be affected by other elements once iron melts to form the alloy, including other light elements. The obtained maximum concentration of H (*x* < 0.4) in the present study gives the lower limit. No matter whether the iron was molten or solid, water was an essential constituent in the evolution of early Earth and promoted the reaction between Fe and S and the dissolution process of the latter.

## Methods

Defined starting materials were used for simulations of the ideal composition of the primitive Earth, namely, the “Fe–S–MgSiO_3_–H_2_O system”. Table 1 summarizes the initial compositions of four types of sample classified in terms of the existence of water and/or S–hydrous samples with/without S and anhydrous samples with/without S. Iron powder was mixed with about 5–10 wt% of sulfur powder in accordance with the S composition of Earth’s core given in previous studies (e.g.^45^). In some experimental runs, no S powder was added into Fe for comparison. The powders were pelletized to an appropriate size (2.0 mm in diameter and 1.2 mm in height) and placed in the center of the sample capsule. A powder mixture of quartz (SiO_2_) and deuterated brucite Mg(OD)_2_ (1:1 molar ratio for saturated water in the system to give a hydrogen concentration of *x* =  ~ 1.0 in FeD_*x*_) surrounded the Fe (+ S) pellet to avoid contamination of carbon from the graphite sample capsule into Fe. Deuterated substitute (Mg(OD)_2_) was used to reduce the high background originating incoherent scattering of H in diffraction patterns. No obvious isotope effect on dehydration reactions was observed between the experiments using two different H/D sources of Mg(OH)_2_ and Mg(OD)_2_. In other experimental runs, MgO was used instead of Mg(OD)_2_ for investigating anhydrous samples as a water-free system. High-PT experiments were performed using the six-axis multi-anvil press “ATSUHIME”^19^ installed at PLANET beamline (BL11)^18^, MLF, J-PARC, Tokai, Japan. We improved the multi-anvil 6–6-type-cell assembly^17^ as described below. Second-stage anvils with a smaller truncation edge length (TEL) of 7 mm were used to achieve pressures above 10 GPa. Anvils with TEL = 10 mm were used for experiments at pressures of up to 6–7 GPa. The cell assembly for anvils of TEL = 7 mm is shown in Supplementary Fig. S2. A dual sample capsule composed of graphite (inner) and NaCl (outer) was used to effectively seal D and water (D_2_O) produced under high-PT conditions. Pressure was determined from the lattice parameters of the NaCl sample capsule based on the equation of state for NaCl^46^. Graphite was applied as the heater, and temperature was estimated from the electric power applied to the heater based on the power–temperature relationship determined using a dummy cell with a Pt–Pt_87_Rh_13_ (0.2 mm in diameter) or W_97_Re_3_–W_75_Re_25_ (type-D, 0.13 mm in diameter) thermocouple beforehand. The temperature fluctuation was estimated within ~ 3 K.

Samples were pressurized up to 6–7 or 10–12 GPa at room temperature and then heated stepwise to 1000–1200 K by 50–100 K per minute. The phase transformations of Fe, dehydration of Mg(OD)_2_, and formation of FeS and silicates were carefully checked in real time. The incident beam size was adjusted to 1 mm × 1.5 mm in width and height (for > 10 GPa) or 1 mm × 2 mm (for 6–7 GPa) to obtain the diffraction patterns deriving exclusively from Fe phases. After compression and subsequent heating, the position of the sample was scanned by 0.5 mm while quickly measuring the diffraction patterns (for less than 10 s) to optimize the position of the sample (Fe). The diffraction data obtained over a long period of time (5–10 h) were refined by the Rietveld method^47^ using GSAS^48^ to determine the D concentration and structural parameters of FeD_*x*_ polymorphs and FeS. The range in *d* for the refinements was from ~ 0.5 to ~ 3.0 Å. To determine ∆*V*(D), the volume of pure Fe was calculated using the equations of state obtained by Tsujino et al*.*^49^ for *fcc* Fe and Fei et al*.*^50^ for *hcp* Fe. As the deuteration reaction is known to proceed during long-duration measurements of > 10 h^17^, we double-checked by comparing the data for the whole exposure time with those for the last couple of hours. During the long-duration measurements, the pressures remained almost unchanged, showing a variation of less than ± 0.1 GPa.

All of the samples that were recovered from neutron diffraction measurements were cut into halves and carefully polished without using water. They were analyzed using an X-ray diffractometer equipped with a position-sensitive proportional counter (PSPC, Rigaku; 40 kV and 200 mA; CrKα) and a scanning electron microscope (SEM) equipped with an energy-dispersive X-ray (EDX) spectrometer (JEM IT-100, JEOL) at ISSP, the University of Tokyo, Tokyo, Japan. The existence of water in silicates of the recovered samples was also examined from infrared (IR) absorption spectra recorded on a Fourier transform IR spectrometer (INVENIO, Bruker) at GCRC, the University of Tokyo. The silicate powders were mixed with KBr diluent and mounted as disks for the analyses.

## Supplementary Information

Supplementary Information.
