# Supplementary material for: Behavior of light elements in iron-silicate-water-sulfur system during early Earth’s evolution
Source: Sci Rep. 2021 Jun 24;11:12632. doi: 10.1038/s41598-021-91801-3 (PMC8225640; doi:10.1038/s41598-021-91801-3)
Supplement: Supplementary file 1 — Supplementary Information. [file 41598_2021_91801_MOESM1_ESM.pdf]

Supplementary Information for

Behavior of light elements in iron-silicate-water-sulfur system during early Earth's evolution

Riko Iizuka-Oku*, Hirotada Gotou, Chikara Shito, Ko Fukuyama, Yuichiro Mori,

Takanori Hattori, Asami Sano-Furukawa, Ken-ichi Funakoshi, Hiroyuki Kagi

*Corresponding author (Riko Iizuka-Oku): riizuka@eqchem.s.u-tokyo.ac.jp

The PDF file contains:

Supplementary Figures S1, S2

Supplementary Table S1

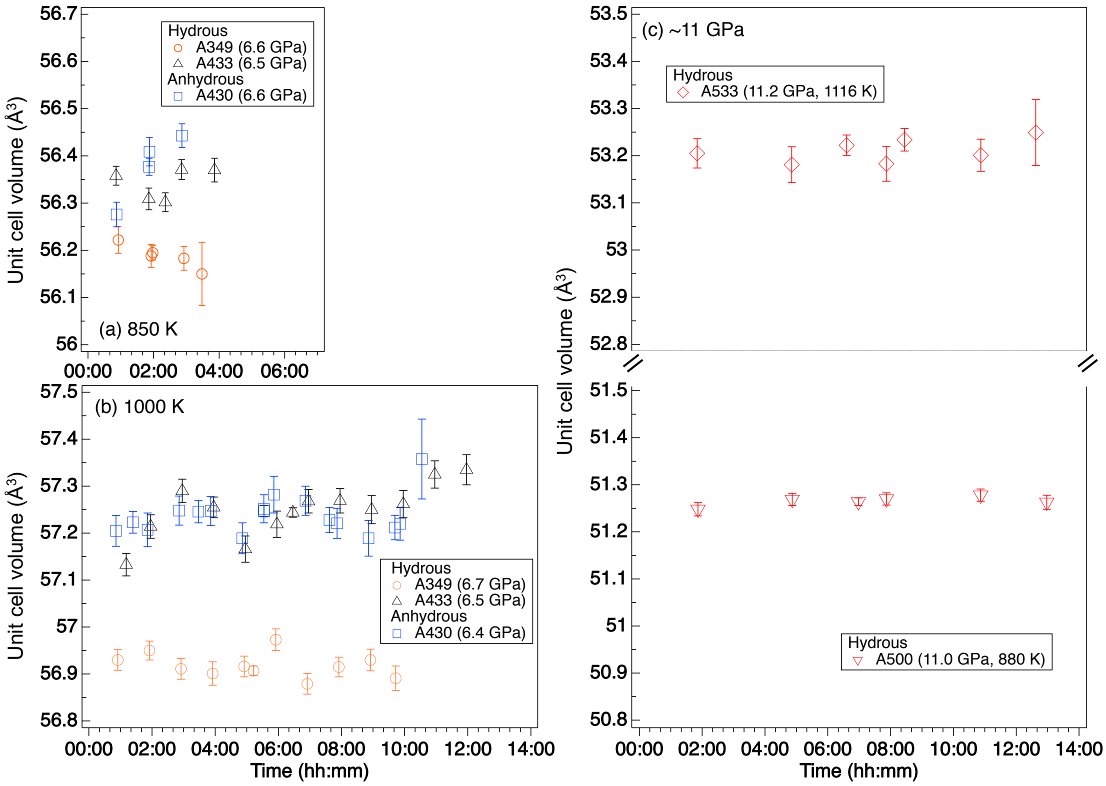

Supplementary Figure S1.

Volume change in the FeS high-P phase (i.e., FeS–V) with time **(a)** at temperature of 850 K, **(b)** at temperature of 1000 K, and **(c)** at pressure of ~11 GPa. No systematic volume expansion was observed during the long-duration measurements (~10 h) regardless of pressure, temperature, and the presence of water.

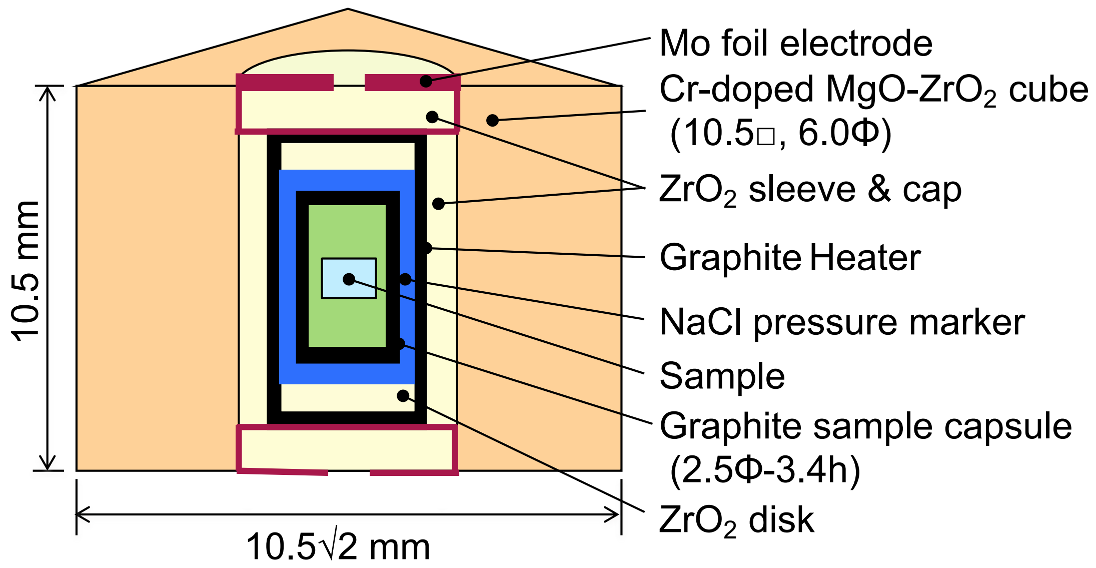

Supplementary Figure S2.

MA6-6 cell assembly for second-stage anvils with a truncation edge length (TEL) of 7 mm. A dual sample capsule of NaCl and graphite was used to seal both dehydrated water and H at high-PT. Iron or Fe + S was placed in the center of the sample capsule and surrounded by silicate to avoid reaction with the graphite capsule.

Supplementary Table S1. Results of Rietveld refinement analysis. Starting material, run#, P–T, duration, observed Fe phases [Fe (*fcc* and *hcp*), FeS–V, and FeO] and their lattice parameters, volume, deuterium content (x) and U_{iso}, and site occupancy.

Sample	Run#	P (GPa)	T (K)	Fe phases	***Fe*** a (Å)	c (Å)	V ($Å^3$)	gD(O)	gD(T)	D content x	U_{iso} Fe ($100×Å^2$)	U_{iso} D ($100×Å^2$)	R_{wp} (%)	R_p (%)	**FeS-V** a (Å)	c (Å)	V ($Å^3$)	a/c	**FeO** a (Å)	V ($Å^3$)
Fe, $Mg(OD)_2+SiO_2$	A429	6.7	850	fcc	3.5813 (2)		45.933 (9)	0.12 (18)	0.009 (14)	0.14 (3)	2.6 (3)	4.5 (18)	7.67	7.21					4.3043 (9)	79.75 (5)
		6.5	1000		3.6165 (3)		47.302 (11)	0.2 (2)	0.021 (19)	0.21 (3)	1.05 (8)	3.7 (8)	6.31	6.01					4.3106 (6)	80.10 (4)
	A445	11.0	746	hcp	2.5432 (2)	4.1302 (10)	23.134 (5)	0.2 (2)		0.17 (3)	1.0 fixed	3.5 (12)	14.2	10.3					4.2655 (8)	77.61 (4)
		11.2	1067	fcc	3.6526 (4)		48.730 (16)	0.2 (2)	0.07 (2)	0.33 (4)	0.79 (2)	5.2 (13)	8.82	7.46					4.2753 -	78.145
	A454	11.9	868	hcp	2.5227 (4)	4.0923 (17)	22.555 (7)	0.1 (2)		0.10 (3)	1.08 (6)	4.3 (6)	7.86	6.96					4.2602 (4)	77.32 (3)
				fcc	3.5669 (2)		45.381 (6)	0.19 (14)	0.028 (14)	0.25 (2)	1.08 (6)	4.3 (6)								
		11.6	1177		3.6205 (2)		47.458 (9)	0.21 (19)	0.044 (17)	0.30 (3)	1.00 (7)	4.8 (7)	13.5	10.6					4.2771 (6)	78.24 (3)
	A534	10.7	1116		3.6036 (2)		46.798 (7)	0.16 (14)	0.030 (13)	0.22 (2)	0.71 (6)	4.1 fixed	9.25	8.24					4.2811 (6)	78.46 (3)
**Fe+S**, $Mg(OD)_2+SiO_2$	A349	6.6	850	fcc+FeS	3.5882 (2)		46.197 (4)	0.03 (11)	0.025 (8)	0.08 (2)	1.7 (2)	5 (3)	8.29	7.13	3.4033 (6)	5.6021 (15)	56.195 (15)	1.646	4.3045 (5)	79.76 (3)
		6.7	1000		3.6060 (7)		46.888 (3)	0.1 (2)	0.032 (15)	0.12 (3)	1.8 (1)	5 (3)	6.51	5.95	3.4181 (3)	5.6242 (10)	56.905 (9)	1.645	4.3083 (3)	79.97 (2)
	A433	6.5	850		3.5781 (3)		45.812 (10)	0.08 (14)	0.009 (11)	0.09 (2)	1.6 (2)	5 (2)	6.54	6.15	3.4052 (6)	5.607 (2)	56.30 (2)	1.647	4.3038 (10)	79.72 (6)
		6.5	1000		3.6059 (3)		46.888 (12)	0.1 (2)	0.013 (18)	0.15 (3)	1.2 (2)	3.8 (13)	7.49	7.19	3.4251 (4)	5.6345 (10)	57.244 (10)	1.645	4.3114 (5)	80.14 (3)
	A533	11.2	1116		3.6060 (2)		46.890 (9)	0.16 (13)	0.028 (12)	0.21 (2)	1.0 (2)	2 (3)	21.4	16.8	3.3410 (9)	5.504 (2)	53.20 (2)	1.647	4.275 (5)	78.1 (4)
	A500*	11.0	~880	hcp+fcc+FeS	2.5269 (3)	4.0885 (11)	22.608 (5)	0.1 (2)		0.10 (2)	0.21 (5)	4.0 (14)	9.70	8.53	3.3038 (3)	5.4233 (9)	51.264 (8)	1.642	4.2661 (10)	77.64 (6)
					3.5728 (9)		45.61 (4)	0.14 (14)	0.062 (13)	0.26 (2)	0.21 (5)	4.0 (14)								
**Fe+S**, **MgO**$+SiO_2$	A430	6.6	850	fcc	3.5749 (2)		45.688 (8)	-	-	0	1.8 (2)		11.4	10.6	3.4083 (10)	5.603 (2)	56.375 (19)	1.644	4.3048 (9)	79.78 (5)
		6.4	1000		3.6123 (1)		47.134 (5)	-	-	0	3.1 (3)		6.78	6.15	3.4254 (5)	5.6339 (13)	57.247 (12)	1.645	4.3115 (6)	80.15 (4)
Fe, **MgO**$+SiO_2$	A453	11.8	700	hcp	2.4881 (1)	4.0229 (4)	21.568 (2)	-	-	0	1.20 (10)		11.3	10.1	-				4.243 (8)	76.4 (5)
		11.4	1177	fcc	3.5883 (2)		46.201 (8)	-	-	0	2.29 (11)		5.79	5.65	-				4.2734 (13)	78.04 (7)

Note: *Blowout occurred during the long-duration experiment in #A500. Isotropic atomic displacement parameters (U_{iso}) for the same atomic species (D and Fe) were constrained to be identical. The content of D, x, was calculated from site occupancies of the octahedral and tetrahedral sites of deuterium, gD(O) and gD(T). R_{wp} and R_p are reliability factors (%).
